# riboFrame: An Improved Method for Microbial Taxonomy Profiling from Non-Targeted Metagenomics

**DOI:** 10.3389/fgene.2015.00329

**Published:** 2015-11-17

**Authors:** Matteo Ramazzotti, Luisa Berná, Claudio Donati, Duccio Cavalieri

**Affiliations:** ^1^Dipartimento di Scienze Biomediche Sperimentali e Cliniche, Università degli Studi di FirenzeFirenze, Italy; ^2^Unidad de Biología Molecular, Institut Pasteur de MontevideoMontevideo, Uruguay; ^3^Centre for Research and Innovation, Fondazione Edmund MachSan Michele all’Adige, Italy

**Keywords:** 16S rDNA gene, community profiling, metagenomics, non-targeted approach, short reads, variable region

## Abstract

Non-targeted metagenomics offers the unprecedented possibility of simultaneously investigate the microbial profile and the genetic capabilities of a sample by a direct analysis of its entire DNA content. The assessment of the microbial taxonomic composition is frequently obtained by mapping reads to genomic databases that, although growing, are still limited and biased. Here we present riboFrame, a novel procedure for microbial profiling based on the identification and classification of 16S rDNA sequences in non-targeted metagenomics datasets. Reads overlapping the 16S rDNA genes are identified using Hidden Markov Models and a taxonomic assignment is obtained by naïve Bayesian classification. All reads identified as ribosomal are coherently positioned in the 16S rDNA gene, allowing the use of the topology of the gene (i.e., the secondary structure and the location of variable regions) to guide the abundance analysis. We tested and verified the effectiveness of our method on simulated ribosomal data, on simulated metagenomes and on a real dataset. riboFrame exploits the taxonomic potentialities of the 16S rDNA gene in the context of non-targeted metagenomics, giving an accurate perspective on the microbial profile in metagenomic samples.

## Introduction

Recent years have witnessed the application of next generation sequencing (NGS) technologies to microbial community analyses, providing for the first time information on the taxonomic composition of microbial communities from a variety of different environments, the most noticeable being the human body.

The consolidated strategy for microbial profiling is to apply NGS on target regions of the 16S rDNA gene, the reference molecular marker for prokaryotes (Woese and Fox, 1977; De Filippo et al., 2010). Despite their power, it has recently been shown that targeted approaches might introduce sequencing artifacts, due to unbalanced amplification (Engelbrektson et al., 2010) or the formation of chimeric amplicons (Haas et al., 2011) or biases due to the inability of the universal primers to evenly amplify the 16S variable regions at all taxonomic ranks (Hamady and Knight, 2009). In addition, a further source of bias is introduced by the limited read length of NGS technologies that does not allow sequencing of the full 16S rDNA gene (Conlan et al., 2012).

Non-targeted metagenomics uses NGS techniques to sequence the whole genome content of an environmental sample and therefore does not depend on prior target selection (Riesenfeld et al., 2004; De Filippo et al., 2012; Dark, 2013). Such techniques are less affected by amplification biases, since they generally rely on less PCR cycles with perfect universal primers. Despite this, highly divergent GC content of the inserts may inherently show a different amplification efficiency, so recent amplification-free protocols or other modifications have been proposed. Although the primary use of non-targeted approaches is the profiling of the metabolic potential of microbial communities, they can also be used to assess relative species abundance using heuristic searches against reference genomes or other sequence databanks such as the NCBI non-redundant database (Segata et al., 2012; Huson and Weber, 2013). However, genome sequence databanks are based on a limited, although growing, number of organisms for which a genome has been entirely sequenced, giving an inherent bias to microbial profiling. A second drawback is that often genome information for unknown or novel genes is incomplete or error prone, due to the limitations in several of the sequence assembly tools available for large-scale NGS data (Vázquez-Castellanos et al., 2014).

Recently, several tools have been developed to identify ribosome-associated reads in non-targeted metagenomic samples, exploiting the constantly increasing coverage of the entire microbial kingdom provided by 16S rDNA databanks such as RDP (Cole et al., 2013), GreenGenes (DeSantis et al., 2006) or SILVA (Quast et al., 2012). These tools use profile stochastic context-free grammars (Nawrocki et al., 2009), Burrows–Wheeler indexing (Li and Durbin, 2010), BLAST-like heuristics or hidden Markov models (Hartmann et al., 2010; Lee et al., 2011). The main aim of these algorithms is to identify reads of ribosomal origin and remove them from metagenomics datasets, in order to facilitate the functional analysis of the remaining reads. No explicit use of these ribosomal reads is generally implemented or suggested.

A new tool named EMIRGE was developed (Miller et al., 2011) with the aim of reconstructing full-length 16S rDNA genes from metagenomes using recruitment and avoiding assembly (being the assembly of the 16S rDNA gene inherently difficult because it contains highly conserved regions mixed to extremely variable regions). Ribosomal reads are recruited by mapping on a 16S gene dataset and then the mapping is iteratively refined with Bayesian expectation-maximization, until full-length 16S genes have been associated to a set of reads. However, this approach heavily relies on the accuracy and completeness of the reference databases and therefore risks to converge to fairly uncharacterized genes, with limited significant improvement of the resolution of taxonomic profiling.

In this work, we introduce riboFrame, a novel method that combines optimized read recruitment with naïve Bayesian classification to provide an automatic, database-free system for microbial abundance analysis in non-targeted (so only marginally biased) metagenomics datasets. Our tool efficiently identifies ribosomal reads from metagenomic datasets and associates them to a position onto the 16S rDNA genes, leaving the user with the possibility to select the different regions of the 16S gene to be used for the taxonomic characterization of the sample. Since riboFrame does not attempt to reconstruct full-length sequences of the 16S rDNA genes, the taxonomic profiling obtained from the different variable regions can be studied separately and compared, giving the opportunity to use non-targeted metagenomic dataset as pre-screening for more focused targeted approaches. The method has been applied on simulated and real datasets demonstrating that riboFrame is a fast, efficient and intuitive tool that provides an accurate, 16S-based microbial taxonomy characterization from non-targeted metagenomic data.

## Materials and Methods

### Description of the riboFrame Procedures

The riboFrame pipeline is composed of two perl scripts (riboTrap and riboMap) and two widely used programs. The final goal is to map Illumina short reads on the 16S gene and then target rank abundance estimates from otherwise non-targeted metagenomic sequencing.

**FIGURE 1:**
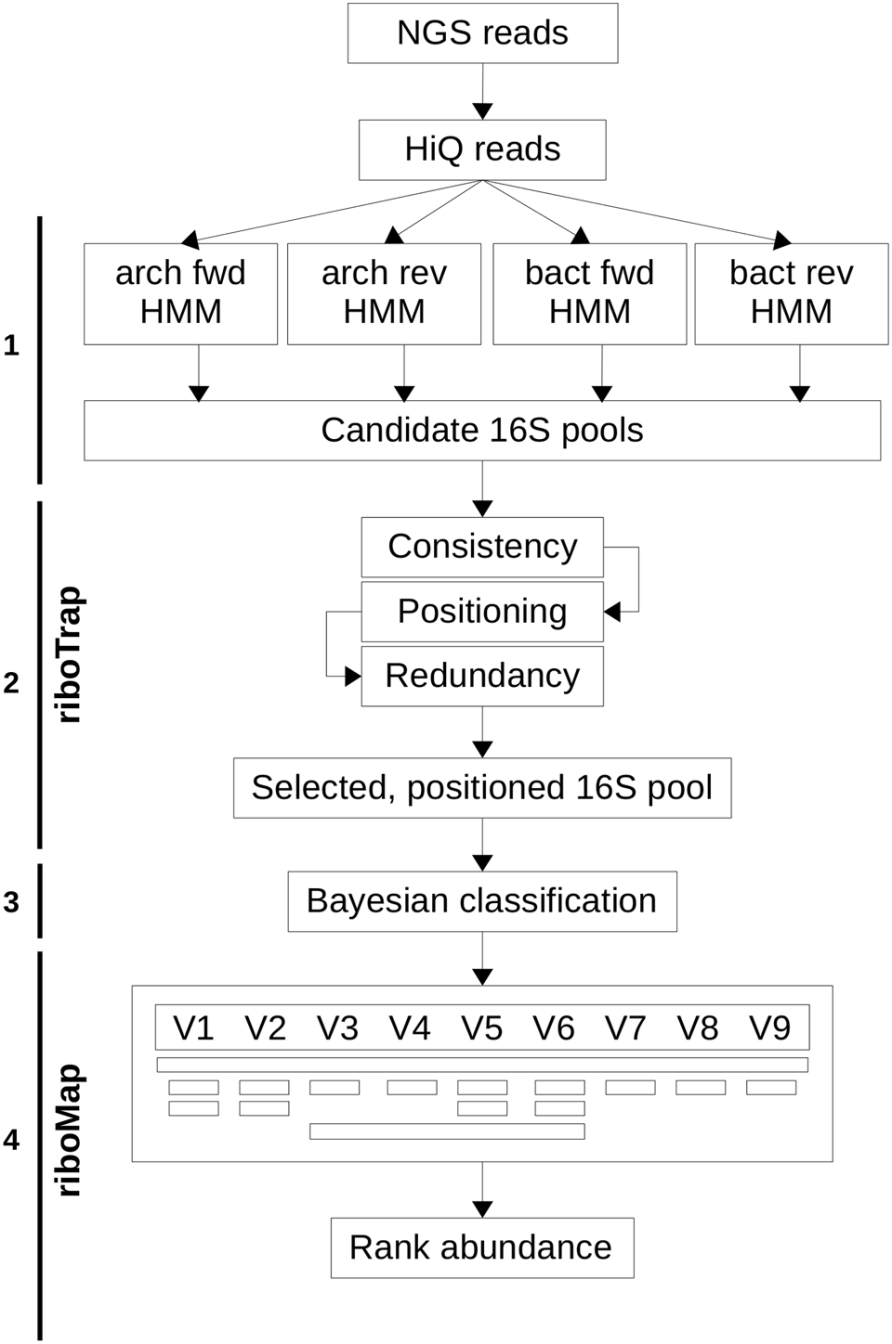
**Scheme of the riboFrame.** After QC of next generation sequencing (NGS) reads, the hmmsearch (HMMER3) is used to identify 16S ribosomal reads in both bacteria and archaea, using HMMs developed in rRNAselector (step 1). The riboTrap program then filters out incongruent assignments and de-replicate multiple assignments in order to create a set of accurate 16S reads supplemented with positional information (step 2). 16S reads are then classified using RDPclassifier to obtain a full domain to genus classification (step 3). The riboMap program eventually filters reads according to rules specified by the user, with a flexible and intuitive scheme, and performs the final rank abundance analyses (step 4). For a detailed description see the section “Materials and Methods – Description of the riboFrame Procedures.”

As depicted in **Figure 1**, the riboFrame pipeline starts after raw Illumina data have been pre-processed for quality control and the procedure involved four steps that will be hereinafter described.

(1) Identification. The hmmsearch command from the HMMER3 package (Eddy, 2011) is issued separately on reads files (single end or paired end) using the HMMs for 16S rDNA gene of bacteria and archaea developed in the rRNAselector (Lee et al., 2011) project. The *E*-value threshold is set to 1E-5, according to specifications in rRNAselector, all other parameters are left to their default values. The program emits, for each file, several tables with identifiers of reads associated to 16S rDNA and, among others, the position of matching on the model.
(2) Preparation. The riboTrap script elaborates the results of hmmsearch, performing a quality control (minimal length, multiple assignment, coherent strand positioning, *E*-value) and preparing non-redundant fasta-formatted files for further processing. Fasta headers are reformatted to include the position of the read in the 16S model. riboTrap also measures the coverage of the 16S gene achieved by the extracted reads and optionally creates coverage plots using functions from the graphics package of the R statistical environment.
(3) Taxonomic assignment. A classification is performed on the 16S ribosomal reads using the local version of RDPclassifier (current version 2.10.1) from the Ribosomal Database Project (Wang et al., 2007) that emits, for each read, a full domain-to-genus classification with bootstrap-based confidence values for each called taxonomic rank.
(4) Selection/abundance analysis. The riboMap script elaborates the output of RDPclassifier and, according to user criteria and targets, builds abundance calculations for each taxonomic rank (optionally creating barplots for immediate evaluation of the results). User criteria include thresholds for assignment confidence and for abundance levels. A scoring scheme have been introduced to avoid over-fitting in case of paired end data. For single end data, each read receives a weight of 1. In case of paired end reads, the increase of abundance is weighted at each specific rank: if just one pair is recruited as ribosomal, it is considered a singleton and weighted 1 as in single pair. If both pairs have been recruited as ribosomal, their weight is decreased to 0.5 so that their combined weight is 1 only if they converge to the same assignment. It should be underlined that the possibility of having both reads recruited as ribosomal is a rare event since the 16S rDNA gene length (around 1500 bp) cannot easily accommodate the full length covered by the two reads of 100 bp considering the insert size that frequently averages to 4–500 bp (for a total length of ∼6–700 bp). Variable region targeting is the main feature of riboTrap and is implemented in riboMap. By default, the program considers belonging of a given region a read that contains or is contained in that region, although options are given to alter region boundaries of specific, user defined, amounts (see **Supplementary Figure S1**). The position of variable regions have been hard-coded in the script and can be referenced simply with VX (with X in the 1–9 range) or with position ranges, and a flexible syntax has been thought to facilitate user selection. Once the selection is done, reads outside the target regions are discarded and those in the target are used to compute the abundances at the various ranks using the scoring scheme explained above. Optionally, abundance plots are emitted as well as a coverage plot to verify the efficacy of the targeting.

All the steps indicated above have a processing time that scales linearly with the number of reads and use very little memory. hmmsearch can be parallelized to take full advantage of multi-core processors or other parallelization strategies. This, coupled to the little memory consumption, makes the riboFrame approach very rapid, efficient in resources and and easily scalable. All the experiments described in this work were produced and analyzed on a Lenovo T420 Laptop equipped with an Intel^®^ Core^TM^ i7-2620M CPU at 2.70 GHz and 8 Gb 1333 MHz RAM.

The riboFrame scripts, manuals and detailed instructions are freely available at the riboFrame Project website^1^ or at github (with repository name “matteoramazzotti/riboFrame”).

See supplementary information for a table reporting all the accession codes for the datasets used in this work.

### Simulation of Ribosomal Reads

A dataset of 16S genes for Bacteria and Archaea was obtained from the RDP database in unaligned GenBank format. The files were processed to create associations between individual sequences and complete lineage of the organisms. A perl script (available from the riboFrame websites) was used to randomly extract 100 bp regions from species (strains) belonging to all genera. For creating the “Full” dataset, one read for each species (strains) associated to a genus was extracted, for the “Curated” dataset 100 species per genus were randomly chosen.

### Simulation of Metagenomics Reads

Metagenomics datasets were created using MetaSim (Richter et al., 2008) fed by all NCBI microbial complete genomes and NCBI taxonomy. The taxonomic profile for species selection was arbitrarily built to maintain a proportion between bacteria and archaea of about 10:1. We also filtered organisms to ensure that a full taxonomic classification could be given to each species according to the Bergey’s taxonomic outline (Wang et al., 2007) used by RDPclassifier. The number of genera actually represented in the reads resulted to be 307 and their proportions reflect that of completely sequenced microbial genomes.

Three 100 bp paired-end reads set consisting of 2, 4, and 10 millions of reads (termed 1, 2 and 5 M, respectively) were created using an Illumina-specific read error model from Plantagora^2^. Each read was mapped on the corresponding genome to determine if it was extracted from a ribosomal operon, so that we could build a testing ground for riboTrap evaluation.

### Preparation of the Datasets from Human Microbiome Project

From HMPDACC site, we selected a case study, the stool sample SRS011061, for which the pyrosequencing reads for the 16S V1–V3 region (SRX020621, 9019 reads and SRX020603, 6864 reads) and V3–V5 region (SRX0200622, 8888 reads and SRX020602, 8422 reads), and the pre-processed Illumina paired-ends reads (sample SRS011061 61478987 reads for pair 1, 28606567 reads for pair 2) were downloaded from NBI SRA and HMPDACC, respectively. Both 16S reads and Illumina reads were downloaded as datasets pre-processed according to Human Microbiome Project (HMP) guidelines (please consult the 16S_SOP.pdf and ReadProcessing_SOP.pdf documents available at hmpdacc.org for further details). We then joined the reads targeting the same region to create two main sequence set, namely V1–V3 (14670 reads) and V3–V5 (14734 reads). Their taxonomic classification of pyrosequencing reads was obtained with RDPclassifier (Wang et al., 2007) with a confidence threshold of 80% and an abundance calculation at the different taxonomic ranks was performed. Illumina reads were processed with our riboFrame method, targeting the V1–V3 and V3–V5 regions in order to create results comparable with those obtained with the pyrosequencing experiments.

### Configuration of Other 16S Ribosomal Read Extractors

For Infernal, we obtained calibrated covariance models from RFAM (Gardner et al., 2010) for the 16S gene of bacteria (RF00177) and archaea (RF01959), we then used the Infernal cmscan command for the actual read recruitment with an *E*-value cutoff of 10^-5^ (i.e., the same threshold used in hmmsearch). For V-Xtractor we used default values (including the suggested SSU HMM-specific *E*-values) and we considered as “extracted” all reads that had matches in at least one of the HMMs spanning the flanks of the variable regions, despite their length. For metaxa we used default values (but we excluded pre-clustering with MAFFT) and we considered as of ribosomal origin also sequences attributed to mitochondria and chloroplasts. In all cases, for comparison purposes, we used just 1 CPU core to test the speed of the algorithms, but it should be underlined that all methods can be run in multi-core systems. For EMIRGE, both paired-ends reads of the HMP sample SRS011061 were used using -l 100 -i 300 -s 100 as command line parameters and the SILVA database provided with the program (indexed with Bowtie, according to EMIRGE manual.) as a reference. The abundance was eventually extracted from the “Prior” field of each sequence identifier after the last iteration.

## Results

### Description of the riboFrame Pipeline

The riboFrame pipeline (**Figure 1**) builds upon HMMER (Eddy, 2011), the most efficient HMM-driven engine for sequence search and RDPclassifier, the reference naïve Bayesian classifier for metagenomics (Wang et al., 2007). The procedure starts with the HMMER3 hmmsearch program that, trained with several models of the 16S gene of bacteria and archaea based on curated databases of sequences of 16S rDNA genes aligned using secondary structure models (Lee et al., 2011), captures ribosomal reads from the mass of reads from Illumina metagenomic sequencing. The riboTrap program extracts the ribosomal reads that are then classified (from domain to genus level) using RDPclassifier that emits for each identified rank a bootstrap-based confidence value between 0 and 1.

The second part of the pipeline, riboMap, takes advantage of the localization of the ribosomal reads in the 16S rDNA gene (the topology) integrated by the confidence score from Bayesian classification to optionally include/exclude specific regions (both constant or variable, see **Supplementary Figure S1**) or low accuracy predictions, creating in the end a domain to genus abundance analysis.

**FIGURE 2:**
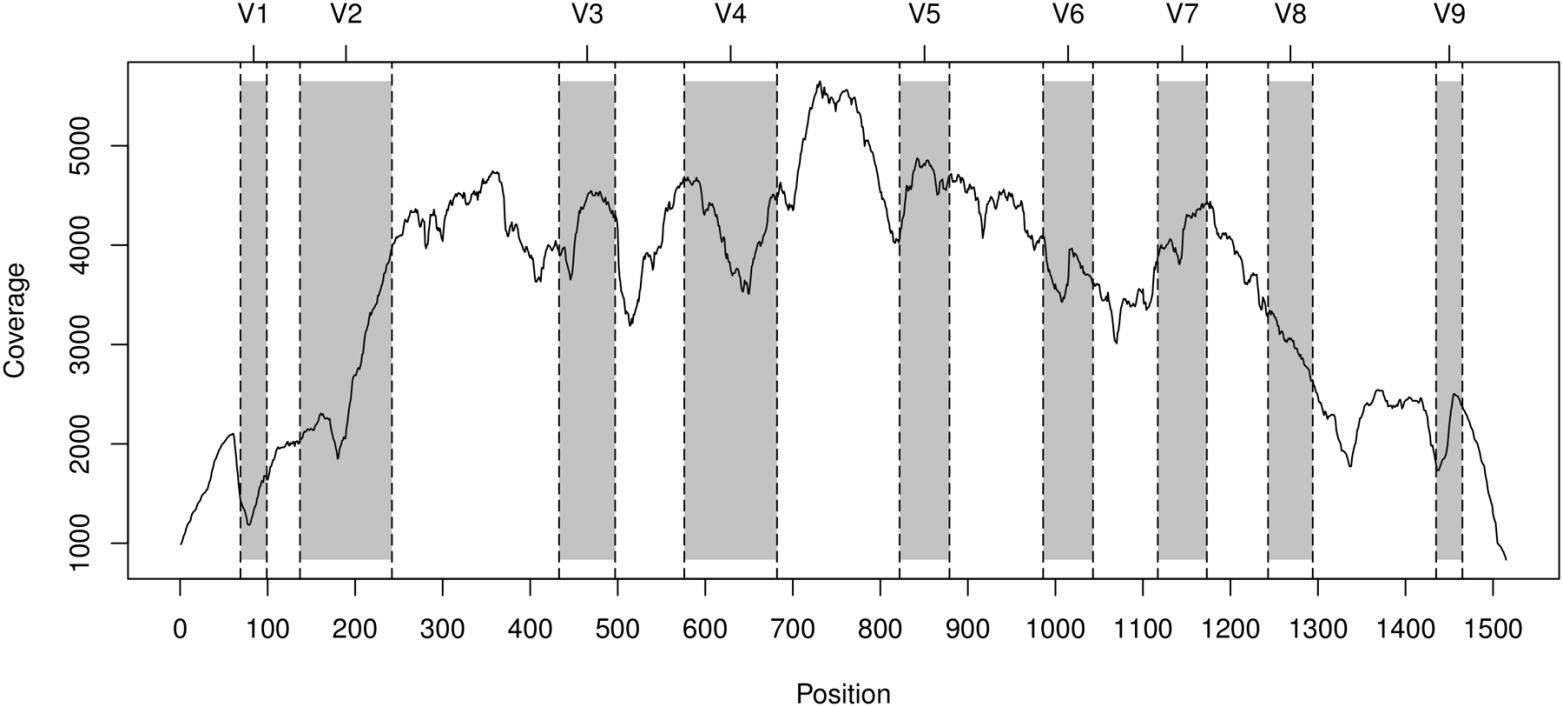
**Coverage of the 16S gene achieved with reads form the Human Microbiome Project (HMP) sample SRS011061 extracted with HMM and topology-annotated by riboTrap.** The trace represent the cumulative coverage for both paired-end reads after riboTrap processing. Shaded areas identify variable regions that are labeled form V1–V9 in the upper horizontal axis.

The coverage of the 16S rDNA gene after region selection can be optionally checked by coverage plots (**Figure 2**) produced by riboTrap, that allows to evaluate whether a sufficient number of reads are available for classification.

### Testing Ribosomal Recruitment with Ribosomal Reads Set

One of the key points to be evaluated concerns the efficiency of recovering ribosomal reads from the pool of non-targeted reads. To assess this aspect we designed two different strategies based on the random extraction of 100 bp reads from the sequences of the 16S genes present in the RDP database (Cole et al., 2013). The RDP database was filtered to include only high quality sequences from prokaryotes that could be unambiguously annotated to the genus rank by the RDPclassifier program according to the Bergey’s Taxonomic Outline of the Prokaryotes (Wang et al., 2007), resulting in a diversity of 1767 bacterial and 103 archeal genera.

Two different sets of reads were extracted. The first set (named “Random”) contained one read per gene of all species assigned to each genus (347174 reads) and was intended to exhaustively explore the recruitment capability of riboFrame. The second, more uniform set (named “Curated”), contained 100 reads per genus selected randomly from sequences classified by RDPclassifier with 100% confidence at the genus level (187000 reads) and was designed to further evaluate the accuracy of the taxonomic classification obtained by riboFrame.

We then evaluated the efficiency of HMMER to align those reads against the 16S HMM for archaea and bacteria (Lee et al., 2011). 308676 reads (88.92%) and 182686 reads (97.69%) were identified as ribosomal for the “Random” and the “Curated” set, respectively (see **Table 1**). This 3–11% loss was found to involve reads located nearby position 200 (using the *Escherichia coli* 16S gene as a reference, see **Supplementary Figure S2A**), possibly indicating that this region (encompassing variable regions 1 and 2) of the HMMs is less accurate or intrinsically more variable. In the “Curated” set we also found that more than 90% of genera had at least 90% reads correctly identified as ribosomal (**Supplementary Figure S2B**), with only slightly reduced performances in the “Random” dataset. No evident signs of biases were present at any taxonomic rank (data not shown) confirming the efficacy of our detection strategy independently from the underlying taxonomic structure. We then proceeded with the classification of the reads with RDPclassifier, according to the riboFrame pipeline. As shown in **Table 2**, we found that the amount of reads classifiable with a 0.8 bootstrap confidence was ∼40%, a percentage closely related to the proportion of the full 16S rDNA gene that is included in the variable V1–V9 regions (i.e., the ones with the highest taxonomic information content), and that the accuracy of the classification was 90.17% on 74110 reads at the genus level (97.38% on 112049 reads at the family level) for the “Curated” dataset and 92.76% on 143287 reads at the genus level (97.10% on 112094 reads at the family level) for the “Random” dataset. These results confirmed that riboFrame can use reads as short as 100 bp to provide a reliable estimate of the taxonomic structure of metagenomic datasets.

**Table 1:** Result of the extraction of ribosomal reads from the simulated datasets “Random” and “Curated.”

	Random	Curated
Original # reads	347174	187000
Extracted by HMM	308686 (88.91%)	182687 (97.69%)
Missed	38488 (11.09%)	4313 (2.31%)


**Table 2:** Results of the evaluation of riboFrame with true ribosomal reads.

	Rank	% Correct	% Wrong	# Reads
Curated
	Domain	100	0	179965
	Phylum	99.91	0.09	166673
	Class	99.62	0.38	156945
	Order	98.92	1.08	137750
	Family	97.38	2.62	112094
	Genus	90.17	9.83	74110

Random
	Domain	100	0	305417
	Phylum	99.97	0.03	293269
	Class	99.88	0.12	283741
	Order	99.14	0.86	248281
	Family	97.1	2.9	193589
	Genus	92.76	7.24	143287


*After ribosomal reads recruitment, riboTrap is used to assign topology to reads and create 16S reads subsets. Such reads are classified with RDPClassifier and compared with the true taxonomy associated to each read. In this case, prediction accuracy is set to 0.8.*

### riboFrame Testing on Simulated Metagenomics Datasets

In order to evaluate the overall performance and accuracy of the riboFrame pipeline we used the MetaSim software (Richter et al., 2008) to build three simulated paired-end metagenomics datasets with increasing size (2, 4, and 10 millions of reads, hereinafter 1, 2, and 5 M, respectively) and a common underlying taxonomic structure containing 1496 species from 307 genera.

As shown in **Table 3**, the initial ribosomal reads screening with HMMER resulted in the detection of 3229, 6248, and 15532 ribosomal reads from the 1, 2, and 5 M dataset, respectively. The observed fraction of ribosomal reads in the pools was 0.15%, in agreement with a grand average estimation of ribosomal DNA proportion in the genomes of prokaryotes (data extracted from the NCBI Genome Database). The average extraction speed of 16S-associated reads was around 2 min 44 s per million of reads (using 4 CPU cores). We obtained, on average, a ∼90% sensitivity and a > 99% specificity for ribosomal reads. Extracted reads were then classified with RDPclassifier and reads in variable regions were isolated with riboFrame (see the coverage plot for the three datasets in **Supplementary Figure S3**). We found that the percent of reads assigned to the correct genus in the three datasets was (on average) 87% at a confidence level of 0.5 (on 30% of the total number of reads) and 95% at a confidence level of 0.8 (on 11.5% of the total number of reads).

**Table 3:** Results of the evaluation of riboFrame with simulated metagenomics datasets.

		Thr 0.5				Thr 0.8			
			
		Good	Error	Reads	Reads%	Good	Error	Reads	Reads%
1M: 3228 reads
	Domain	99.97	0	3209	100.00	99.97	0	3202	100.00
	Phylum	99.59	0.37	2943	91.71	99.95	0	1994	62.27
	Class	99.69	0.27	2568	80.02	99.93	0	1467	45.82
	Order	97.83	2.11	1985	61.86	99.57	0.32	935	29.20
	Family	94.14	5.8	1517	47.27	98.09	1.77	678	21.17
	Genus	88.25	11.64	944	29.42	95.57	4.16	360	11.24

2M: 6247 reads
	Domain	99.95	0.03	6227	100.00	99.97	0.02	6206	100.00
	Phylum	99.75	0.23	5711	91.71	99.95	0.03	3833	61.76
	Class	99.6	0.38	5005	80.38	99.97	0	2872	46.28
	Order	98.38	1.6	3940	63.27	99.84	0.11	1867	30.08
	family	94.62	5.35	2992	48.05	98.54	1.39	1367	22.03
	Genus	88.03	11.92	1895	30.43	95.99	3.87	722	11.63

5M: 15531 reads
	Domain	99.98	0.01	15462	100.00	99.99	0.01	15427	100.00
	Phylum	99.69	0.3	14185	91.74	99.97	0.02	9626	62.40
	Class	99.6	0.4	12381	80.07	99.99	0	6994	45.34
	Order	98.16	1.83	9558	61.82	99.65	0.33	4558	29.55
	Family	94.01	5.98	7158	46.29	98.25	1.72	3318	21.51
	Genus	86.6	13.38	4551	29.43	93.69	6.25	1742	11.29


### A Real Life Metagenomics Dataset from HMP

The performances of riboFrame were further evaluated using publicly available data from the HMP that, for many samples, provides Illumina-based metagenomics paired to microbial profiling with amplicon-based pyrosequencing. These data allow to correlate the taxonomic assignment and abundance estimates obtained from 16S amplicon based metagenomics to the results of methods, like riboFrame, based on non-targeted metagenomics. We selected a sample with known high complexity (SRS011061, a stool sample, since gut is widely accepted as one of the most diverse and rich habitat within the human body), for which the 16S profiling based on the V1–V3 and V3–V5 variable regions of the 16S rDNA gene, as well as Illumina non-targeted metagenomics data were available. We then used riboFrame to build microbial profiles from the latter and then compared the results with the former.

### riboTrap-processed Metagenomic Reads are in Agreement with 16S Targeted Pyrosequencing

**FIGURE 3:**
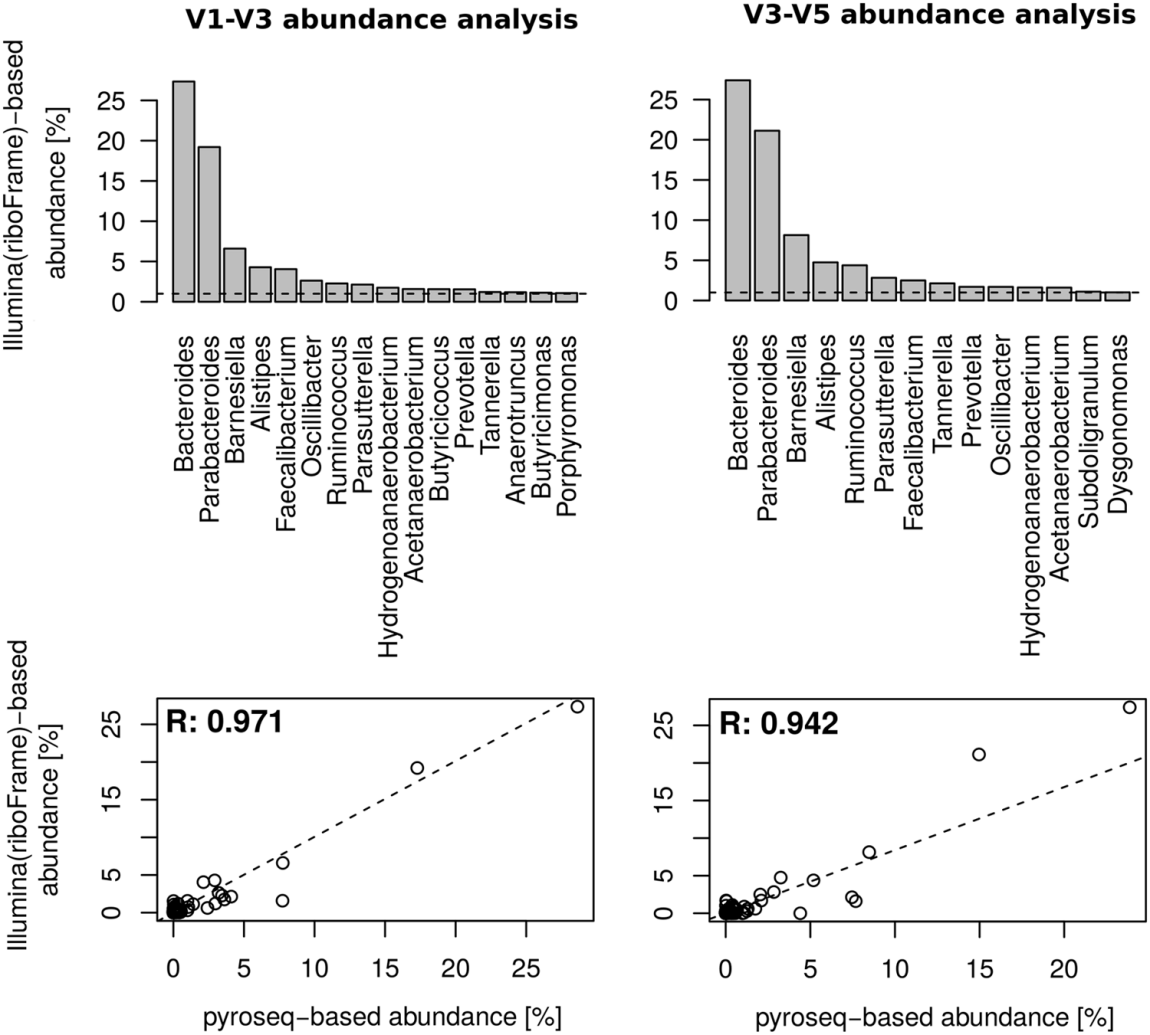
**Comparison of microbial profiling between riboFrame and 16S rDNA pyrosequencing on HMP sample SRS011061.** **(Top)** Barplots of genus-level abundance calculation on two 16S regions targeted by Illumina sequencing after the riboFrame processing. Left and right columns present results from 16S rDNA variable regions V1–V3 and V3–V5, respectively. Only genera accounting for at least 1% of the total classifiable reads are shown. **(Bottom)** Scatterplot depicting the full range of abundances % obtained with pyrosequencing (*x*-axis) and with riboFrame-processed Illumina reads (*y*-axis), along with a linear best fitting line (dashed). The Pearson correlation coefficient (R) of the two dataset is also present.

The hmmsearch/riboTrap procedure extracted a total of 63262 reads identified as belonging to the 16S gene from the pool of Illumina-based meatgenomics reads. The plot in **Figure 2** shows good coverage of the target regions V1–V3 and V3–V5, suggesting that reads overlapping these regions can provide an accurate taxonomic profile of this sample. Ribosomal reads were then classified with RDPclassifier. riboMap identified 17691 reads overlapping the V1–V3 region and 23519 overlapping the V3–V5 region. The rank abundance analysis at 0.8 confidence threshold (shown in **Figure 3**) demonstrated that, although differences existed, an excellent correlation was present at the genus level, the lower rank reachable with RDPclassifier, in the two regions. The correlation coefficient of abundance percent at the genus level in Illumina riboFrame-processed vs. pyrosequencing reads was 0.971 for the V1–V3 region and 0.942 for the V3–V5 region, confirming that riboFrame processing of non-targeted Illumina reads gives results comparable to those obtained with targeted pyrosequencing. As expected, ranks higher than genus resulted in much closer agreement between the two techniques (see **Supplementary Figure S4**).

### Read Length and Confidence in Taxonomic Assignment

In order to evaluate the performance of short reads in microbial classification with the naïve Bayesian methods, we first analyzed how read length affected the confidence of assignments at the different taxonomic ranks. For each rank, and at each read length, we analyzed the three central quartiles to ensure a correct quantification and representation (see the plots in **Supplementary Figure S5**). As expected, at the domain level most reads can be assigned with high confidence (>=0.8) even in reads as short as 60 bp (the minimal size imposed by QC-filters). The phylum-, order- and family level assignment showed a decrease of performances with a reasonable limit to 90 bp. As expected, at the genus level assignment was supported only for reads of maximum length, justifying the filter-by-length option offered by the riboTrap script of the riboFrame pipeline.

To further evaluate the impact of the accuracy confidence limits on the number of reads identified as ribosomal and used in taxonomic classification, we next investigated how the number of accepted reads varied as a function of the increase in confidence score at the different taxonomic ranks. The data reported in **Supplementary Figure S6** clearly show that at the family level, more than 60% of the reads have confidence score >=0.8 while at the genus level the percent decreases to about 45%, a relatively high proportion taking into account that the length of the reads varied from 60 to 100 bp.

### Independent Evaluation of the 16S Variable Regions

The previous analyses on read length did not take into account the fact that confidence is expected to vary along the 16S gene due to the presence of variable (highly informative, poorly conserved) and constant regions (less informative, highly conserved). We then took full advantage of the riboMap capability of inspecting different regions separately. We evaluated the distribution of RDPclassifier confidence scores at the genus rank in the nine variable regions, using a tolerance (i.e., the possibility of enlarging the window for reads recruitment, riboMap “tol” option) of 20% of the variable region length. As shown in **Supplementary Figure S7**, the regions V3–V5 are characterized by a higher proportion of high confidence scores (>=0.8). A large body of literature indicates that different variable regions have different performances and biases toward certain groups of prokaryotes (Chakravorty et al., 2007). These results suggest that the V3 and V5 regions have superior classification ability with respect to others, at least in this sample and for the exemplification purposes of this analysis.

**FIGURE 4:**
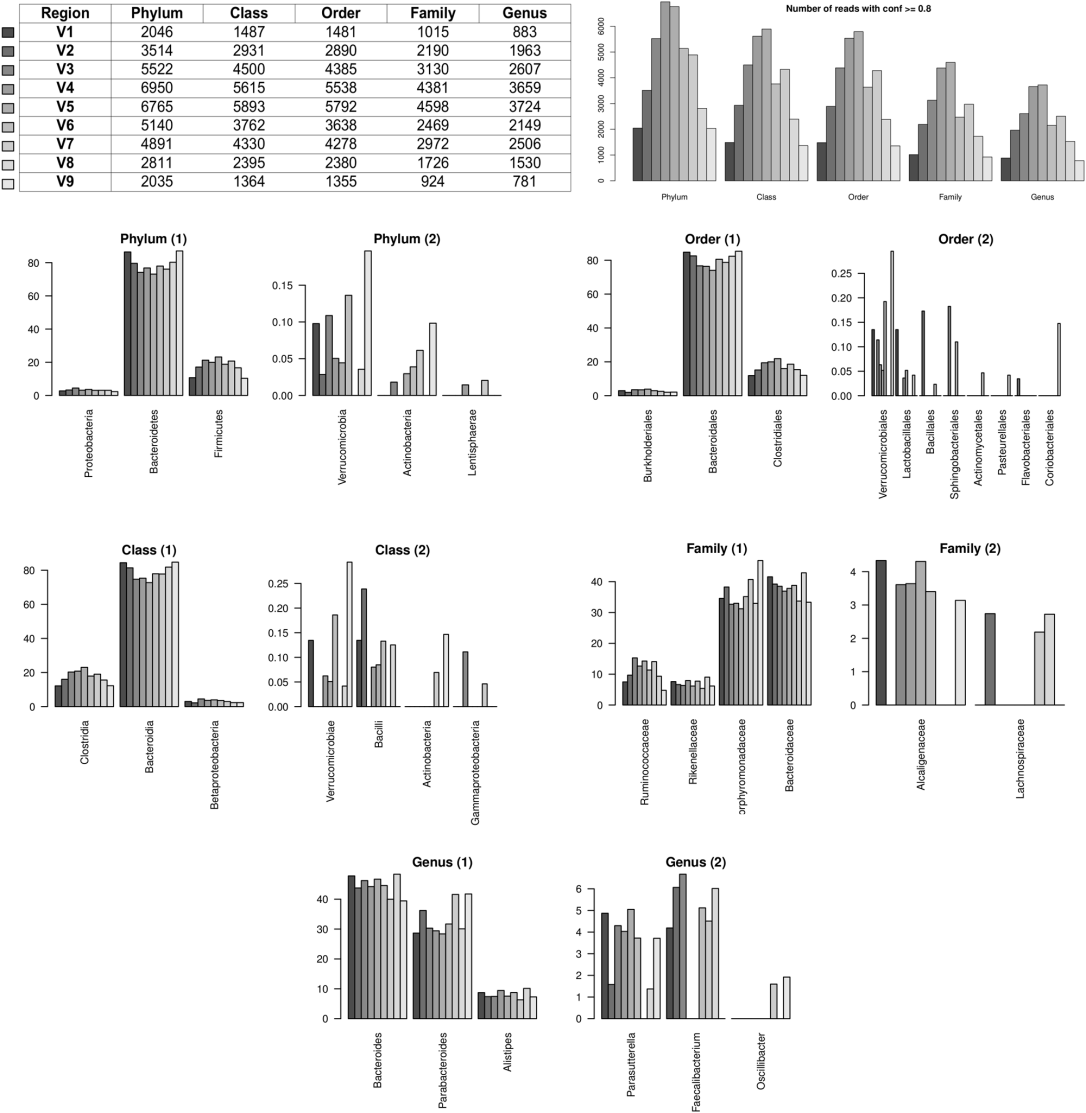
**Taxonomic profiles of the HMP sample SRS011061 obtained with the nine variable regions independently.** The top row shows a table with counts of accepted reads at the various taxonomic ranks in the different regions **(left)**, and a summary barplot showing the same information **(right)**. The following panels shows data obtained by considering only the five most abundant assignments for the different regions at the different taxonomic ranks.

We next evaluated how the taxonomic profiles changed using the different variable regions. We configured riboMap to include reads located in each variable regions separately with a tolerance (see above) of 20% and a confidence score higher than 0.8. We then studied the five most abundant taxonomy assignments for each rank. The results of the abundance analysis are shown in **Figure 4**. Taken together, these results confirm that different regions have different abilities in identifying specific groups of bacteria, reinforcing the idea that an accurate selection of variable regions should be performed before amplicon-based sequencing experiments, especially in the case of studies specifically focused on specific classes of organisms.

### Comparison with Existing Tools

In order to evaluate the performances of the extraction procedure we used the “Curated” dataset (187000 reads) for a comparative test against three previously published systems for identifying ribosomal reads, i.e., Infernal (Nawrocki et al., 2009), based on profile stochastic context-free grammars, metaxa (Bengtsson et al., 2011) and V-Xtractor (Hartmann et al., 2010), both HMM-driven systems. Since these tools are only designed to identify ribosomal associated reads in metagenomic datasets, but do not provide tools for taxonomic profiling, we compare the performances only for the identification step. Moreover, for comparison purposes (and to ensure the feasibility of our approach on average personal computers) the tests were performed on 1 CPU core. Default parameters were used for all programs.

As shown in **Table 4**, V-Xtractor took more than 810 min to complete the extraction of reads and failed to recruit 1.52% ribosomal reads. Metaxa took 525 min and missed 15.20% of the ribosomal reads. Infernal took 2870 min and missed 1.14% of the ribosomal reads. Hmmsearch, optimized for riboFrame, completed the analysis in 30 min, missing 2.31% of the ribosomal reads. These results show that the riboFrame strategy is fast compared to other methods on large metagenomic datasets without significant loss of sensitivity.

**Table 4:** Result of the extraction of ribosomal reads from the “Curated” ribosomal reads set (187000 reads) by various extractors.

	Recruited	Error%	Time (min)^∗∗^
riboFrame^∗^	182687	2.31	30
Infernal	184861	1.14	2860
V-Xtractor	184161	1.52	810
Metaxa	159632	15.20	525


*^∗^riboFrame uses HMMER hmmsearch as extractor.*

*^∗∗^Normalized to 1 CPU core of a Lenovo T420 laptop equipped with and Intel^®^ Core^TM^ i7-2620M CPU at 2.70 GHz and 8 Gb 1333 MHz RAM.*

We then compared the performances of riboFrame to EMIRGE, that estimates the taxonomic structure of metagenomic samples from non-targeted sequencing via reconstruction of the full length 16S rDNA gene using reads recruitment and an expectation-maximization algorithm. EMIRGE took about 150 h to complete the analysis on our sample data set, using the SILVA-derived 16S database provided with the EMIRGE program as a reference. For comparison purposes, the resulting abundance table was compared with abundances obtained from pyrosequencing and riboFrame on the 16S rDNA V3–V5 regions. A shown in **Supplementary Figure S8A** the large majority (>76%) of assignments converged to uncultured bacterial species (classified at the genus level at best), indicating that on our HMP dataset the advantage provided by the extremely time consuming assembly of the full 16S rDNA gene accomplished by EMIRGE to increase the classification resolution of metagenomic samples is limited. At higher ranks ranging from phylum to genus (see genus and family level classification in **Supplementary Figure S8B**), the estimated abundances were fairly similar to those obtained with riboFrame in a fraction of EMIRGE computational time.

## Discussion

In this work we developed and evaluated a method for the microbial profiling of metagenomic samples via classification of 16S-derived reads recruited without explicit reference databases and selected based on their positioning (topology) on the 16S gene. The tool we developed, riboFrame, was designed to identify and position ribosomal reads among the huge number of short reads typical of Illumina-based metagenomic projects and to then proceed with taxonomic classification targeting variable regions of the 16S rDNA gene. The predicted abundances at the different ranks were in agreement with the results obtained from 16S amplicon pyrosequencing, especially if considering abundances above 1–2%. Other HMP samples were also tested, obtaining basically super-imposable results that in all cases confirmed the large agreement between riboFrame derived abundances and those obtained with targeted pyrosequencing (data not shown).

The strategy adopted by riboFrame gives the possibility of deciding *a posteriori* the target region to be used for taxonomic classification. riboFrame provides an accurate taxonomic profiling of datasets produced with the target of characterizing the functional profile of microbial communities, allowing the simultaneous determination of the two in a single experiment. Additionally, using the throughput and multiplexing possibilities of Illumina-based technologies, this tool can be used in all cases when amplicon-based sequencing projects need and unbiased pre-screening of the diversity in the sample before deciding the region to address for taxonomic profiling, since it is known that different regions of the 16S gene have different taxonomic classification potentials and some are more adequate than others for specific families of bacteria present in different environments (Chakravorty et al., 2007).

Our analysis on the taxonomic accuracy of 100 bp reads using the naïve Bayesian classifier showed that this size is sufficient to reach a confident genus assignment only in less than half of the reads. One may argue that this is a major limit of our approach based on short reads. Nevertheless, the sampling capacity of Illumina-based metagenomics proved to be sufficient to describe the microbial profile at the genus level, the lowest rank reachable by the Bayesian method. Considering that the increase of read length is one of the most demanding needs for NGS and that all companies have already improved their technologies to achieve this goal, we strongly believe that our method will be of great relevance also in a near future.

Increasing read length can only increase the number of reads confidently classified at the genus level but does not allow a higher taxonomic resolution (e.g., down to the species level). It has been reported that only full-length genes can be used to push characterization to the species level (Schloss et al., 2009). In fact, the scanning with heuristic methods of 16S rDNA databanks, that contain fully annotated species as well as a larger number of completely unknown species, frequently converges into the latter category, reducing the theoretical possibility of reaching a strain or even species-level resolution. We showed that this kind of issues also affects the most advanced 16S rDNA gene reconstruction method, EMIRGE, that characterized our HMP-derived sample as a population mainly composed of uncultured bacterial species. Being such uncultured bacteria classified at the genus level at best, it is evident that strain-level resolution cannot be achieved effectively using short metagenomics reads and, from this perspective, a genus level characterization can be achieved much more efficiently using the approached we used in riboFrame.

One of the most crucial aspects of the riboFrame data processing is the decoupling of the ribosomal reads from a database-derived source. Our choice of using 16S rDNA HMMs, calibrated to the *E. coli* positions and trained on secondary structure-aware sequence alignments of 16S genes, has two advantages. The first is the coherence in positioning the matching reads on the 16S gene model. This, coupled to the existing information about the position of the variable regions, allows to confidently select reads potentially relevant for taxonomic classification. The second is that we highly reduce errors or ambiguous assignments due to the small size of Illumina reads (around 100 bp), that currently represents a limit for recruiters based on heuristic search. In fact, recruiters may fail to accurately identify the correct source due to the similarity in constant regions among different microbes and to the observation that a single microbe can contain multiple ribosomal operons with different length and composition. It is instead established (and confirmed in this work) that a 100 bp length is sufficient for genus-level assignment with naïve Bayesian classification, thus reinforcing the validity of our strategy.

Several methods have been developed to estimate taxonomy from metagenomics experiments. However, current procedures have inherent limitations that will not likely be solved in the near future. Coding sequence-based methods are still limited by the relatively small (although growing) number of reference microbial genomes. Methods relying on reference 16S rDNA data banks have the opposite drawback of identifying as best hits a majority of uncultured and unknown organisms, a fact that limits their theoretical capability of reaching deep levels of taxonomic resolution. On the contrary, although limited in the taxonomic resolution, Bayesian methods for taxonomy assignment trained on the distribution of *k*-mers of 7–8 bp offer fast assignment based on a robust statistics and provide a bootstrap-based confidence easy to interpret, more broadly applicable and with higher general validity. It is worth stressing that the RDPclassifier we used was trained on full-length 16S rDNA genes. As noticed before (Mizrahi-Man et al., 2013), a retraining of the Bayesian methods with shorter reads offers some advantages in accuracy. Although we did not explicitly take into account such aspects, riboFrame can be easily adapted to accept formats different from that of RDPclassifier or HMMER, the most noticeable example being Infernal for reads recruitment and classify.seqs function in mothur for taxonomic assignment (Schloss et al., 2009).

One on the most important innovations introduced by riboFrame is the possibility of evaluating the classification performances of different regions across the 16S rDNA gene. At the phylum level we observed that the three most abundant bacterial phyla (with abundance filtered to be higher than 2%) i.e., Bacteroidetes, Firmicutes, and Proteobacteria were conserved with little (<10%) variations in the proportions. It is worth noticing the opposite trends of abundances between Bacteroidetes and Firmicutes across regions that seem to indicate that V1 and V9 regions tend to erroneously classify the latter as the former. The proportions proved to be more variable for less abundant phyla (abundance lower than 1%, that was already established to be below the sensitivity threshold usually applied in microbial profiling) with Verrucomicrobia and Actinobacteria absent from the top five list in the V7 region, the latter also absent in the V1, V2, and V4 regions. At the order rank the most abundant assignments were Bacteroidales, Clostridiales, and Burkoleriales, with the same trends described at the phylum level. At the class rank Bacteroidia, Clostridia and Betaproteobacteria contributed most to the profile, with Verrucomicrobia absent in the V2, V3, and V7 regions, Bacilli absent in the V3, V7, Actinoacteria and Gammaproteobacteria only detectable at low levels in the V7 and V9 and in the V3 and V7 regions, respectively. At the family level we found that that the Bacteroidia class is represented as equally composed by Porphyromonadales and Bacteroidaceae, with a good agreement across variable regions. It is interesting to notice here that the top five list did not include Alcaligenaceae in regions V2, V7, and V8 and that Lachnospiraceae were only in the list of V2, V7, and V8. Finally, at the genus level Bacteroides and Parabacteroides and Alistipes were the most abundant with good agreement across regions. Curiously, the *Parasutterella* genus was not in the top five list of V7 region, the *Faecalibacterium* was not in the list of V4, V5, and V9 regions and the *Oscillibacter* genus was only in the list of the V7 and V9 regions.

The most noticeable “caveat” in using the riboFrame method is represented by the possible reduced number of reads recruited as ribosomal by the HMM-based search. This may in fact cause a down-sampling error and, accordingly, a decrease in the accuracy of the abundance analysis. Although in our experience the number of Illumina reads from a typical metagenomics project gives a sufficient number of 16S rDNA associated reads, reducing the number of reads (e.g., by multiplexing/barcoding) may hamper the performance of our approach. To allow the user to evaluate this point, riboTrap provides a coverage plot showing how many reads cover the 16S gene after recruitment. Such coverage plots are important snapshots to evaluate the efficiency of the metagenomics sampling of the 16S ribosomal gene and are intended to assist the user in deciding whether to proceed or not with the taxonomy assignment. In addition, riboMap reports the number of reads selected after imposing thresholds in confidence and length, so the user can easily control the sampling depth of the analysis and decide about the trustfulness of the abundance analysis.

The pipeline we introduced, riboFrame, is a rapid, flexible and intuitive method to identify, select and map ribosomal reads onto the 16S ribosomal gene with the aim of performing taxonomic classification. The possibility given by riboFrame of addressing *post hoc* the region to be analyzed allows the comparison of the taxonomic performance of different variable regions.

The riboFrame approach proved to be fast and effective on simulated datasets. More importantly, the application of our method to a public dataset of targeted 16S and Illumina data showed a substantial concordance on genus assignment between microbial composition assessed through pyrosequencing and Illumina sequencing.

riboFrame represents the first attempt to create a tool for dissecting and evaluating the potentiality of a direct, 16S based taxonomic classification of short reads applied to non-targeted metagenomics.

## Author Contributions

MR conceived the algorithms, wrote the codes, tested the results and drafted the manuscript. LB tested the algorithms, did the alpha testing and critically revised the manuscript. CD contributed to optimize the algorithms and to draft the manuscript. DC conceived the algorithms and did critical assessment of the work.

## Conflict of Interest Statement

The authors declare that the research was conducted in the absence of any commercial or financial relationships that could be construed as a potential conflict of interest.

## Footnotes

1 bioserver2.sbsc.unifi.it/bioinfo/riboframe.html

2 www.plantagora.org
