# Supplementary material for: riboFrame: An Improved Method for Microbial Taxonomy Profiling from Non-Targeted Metagenomics
Source: Front Genet. 2015 Nov 17;6:329. doi: 10.3389/fgene.2015.00329 (PMC4646959; doi:10.3389/fgene.2015.00329)
Supplement: Supplementary file 1 [file Supplemental_Data.PDF]

riboFrame: an improved method for microbial taxonomy profiling from non-targeted metagenomics

Matteo Ramazzotti [1] , Luisa Berná [2] , Claudio Donati [3] and Duccio Cavalieri [3,*]

[1] Dipartimento di Scienze Biomediche Sperimentali e Cliniche, Università degli Studi di Firenze, Firenze, Italy.

[2] Unidad de Biología Molecular, Institut Pasteur de Montevideo, Montevideo, Uruguay

[3] Centre for Research and Innovation, Fondazione Edmund Mach, San Michele all'Adige (TN), Italy.

Supplementary Materials

Supplementary figure S1. Effect of different values passed to the "var" parameter in riboMap. The position of variable regions are hard-coded into the program so that the user can refer to them as e.g. V1 or V5 and intuitively target one or more of them.

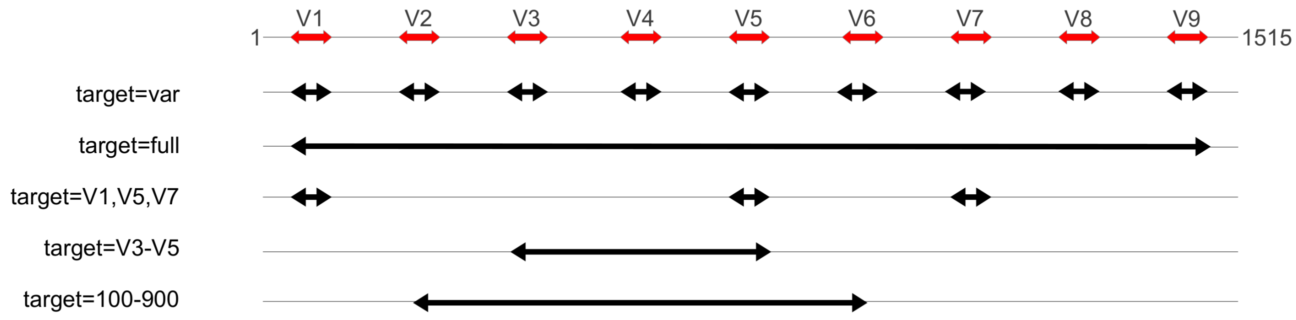

Supplementary figure S2. Results of the analysis on the simulated ribosomal reads. A: 16S rRNA gene coverage of reads extracted from actual 16S rRNA genes and attributed or not to a ribosomal origin by the recruiting system. Green: coverage determined from actual reads (expected). Blue: coverage of reads correctly attributed to the 16S gene (Extracted). Red: coverage of reads incorrectly classified as non-ribosomal (missing). B: performance of the riboTrap extraction system, expressed in terms of percent of reads per genus correctly extracted. Left: "Complete" reads set (347174 reads). Right: "Limited" reads set (187000 reads).

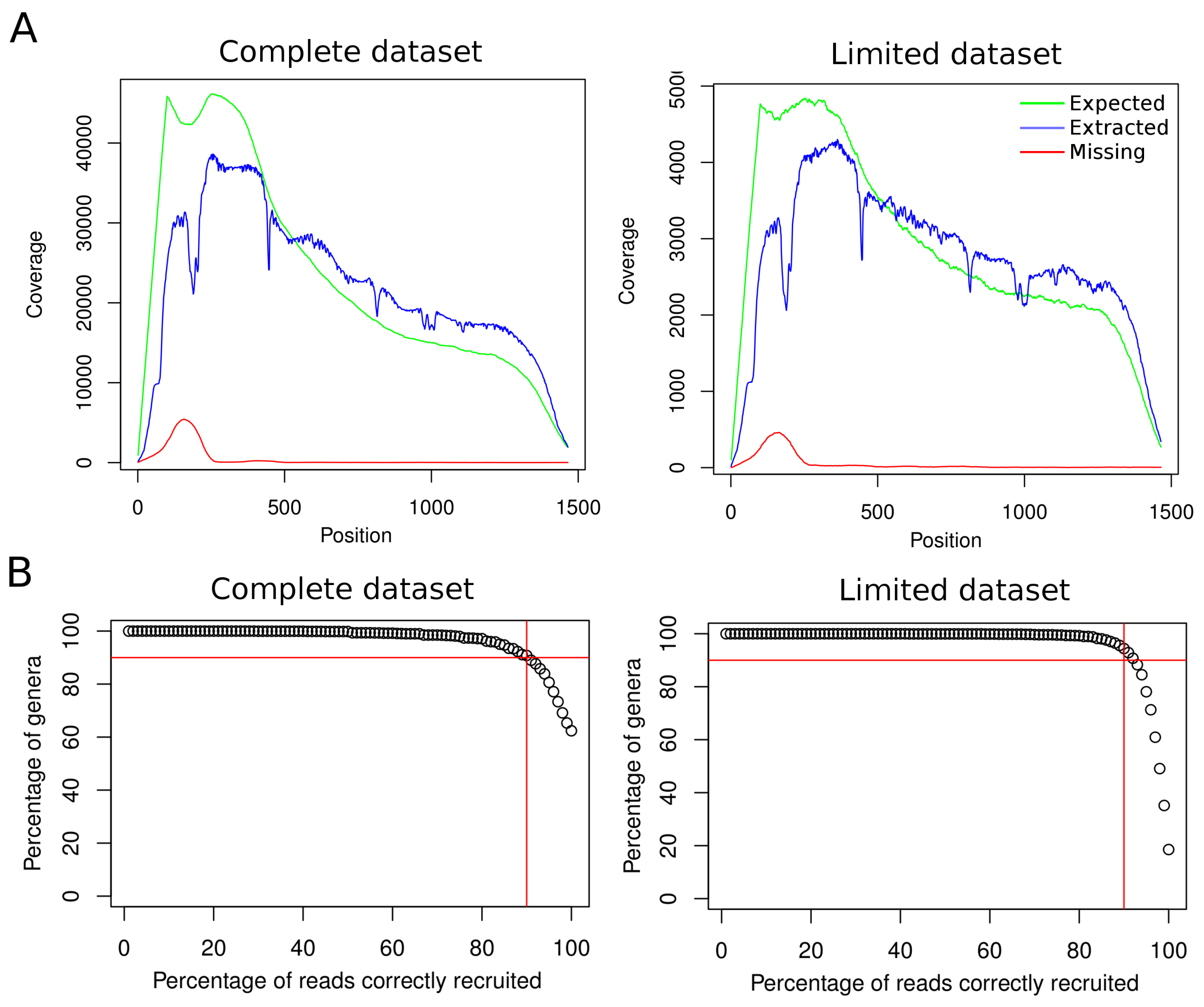

Supplementary figure S3. Coverage plots produced by riboTrap, describing the amount of information extracted from the three simulated metagenomics datasets with equal composition but with different amount of reads, namely 2 millions (1M), 4 millions (2M) and 10 millions (5M).

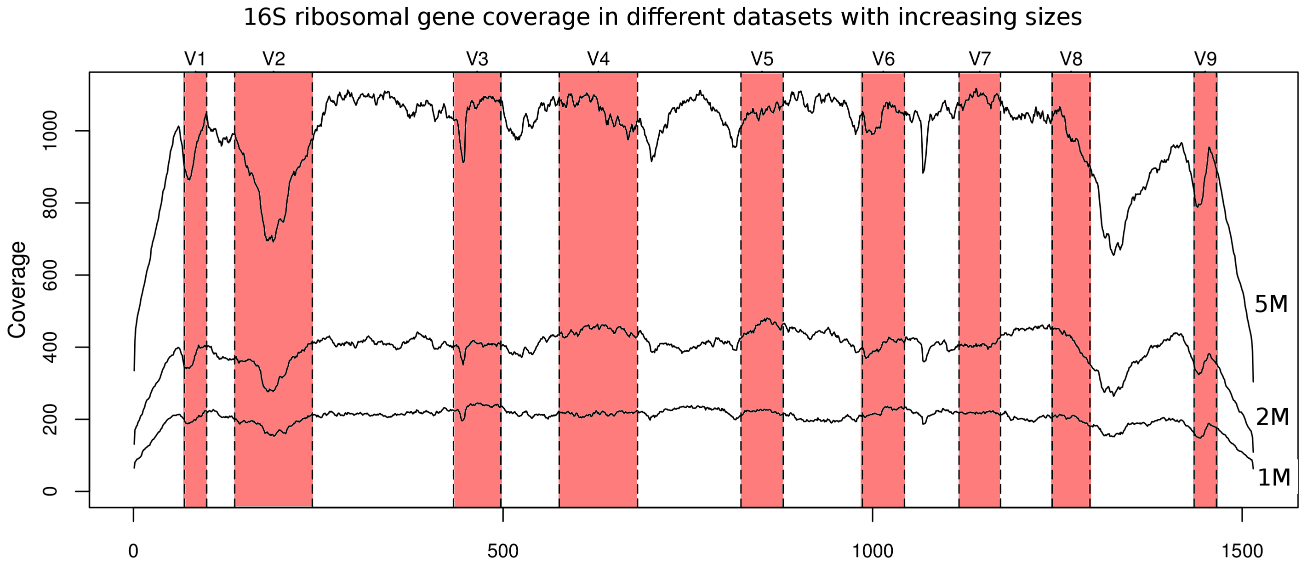

Supplementary figure S4. Rank abundance analysis of the HMP sample SRS011061, comparing the results of pyrosequencing on V1-V3 and V3-V5 regions and the corresponding results obtained from non targeted Illumina sequencing after the riboFrame processing. Here the ranks phylum, order, class and family are depicted, the main text contain results at the genus rank. For readability purposes, the barplots report the abundance % of the specific rank with an 1% cut-off. The scatter plots depict the full range of of abundances % obtained with pyrosequencing and with riboFrame-processed Illumina reads, along with a linear best fitting line (dashed) and the Pearson correlation coefficient (R) and the regression coefficient of the linear model (R^2).

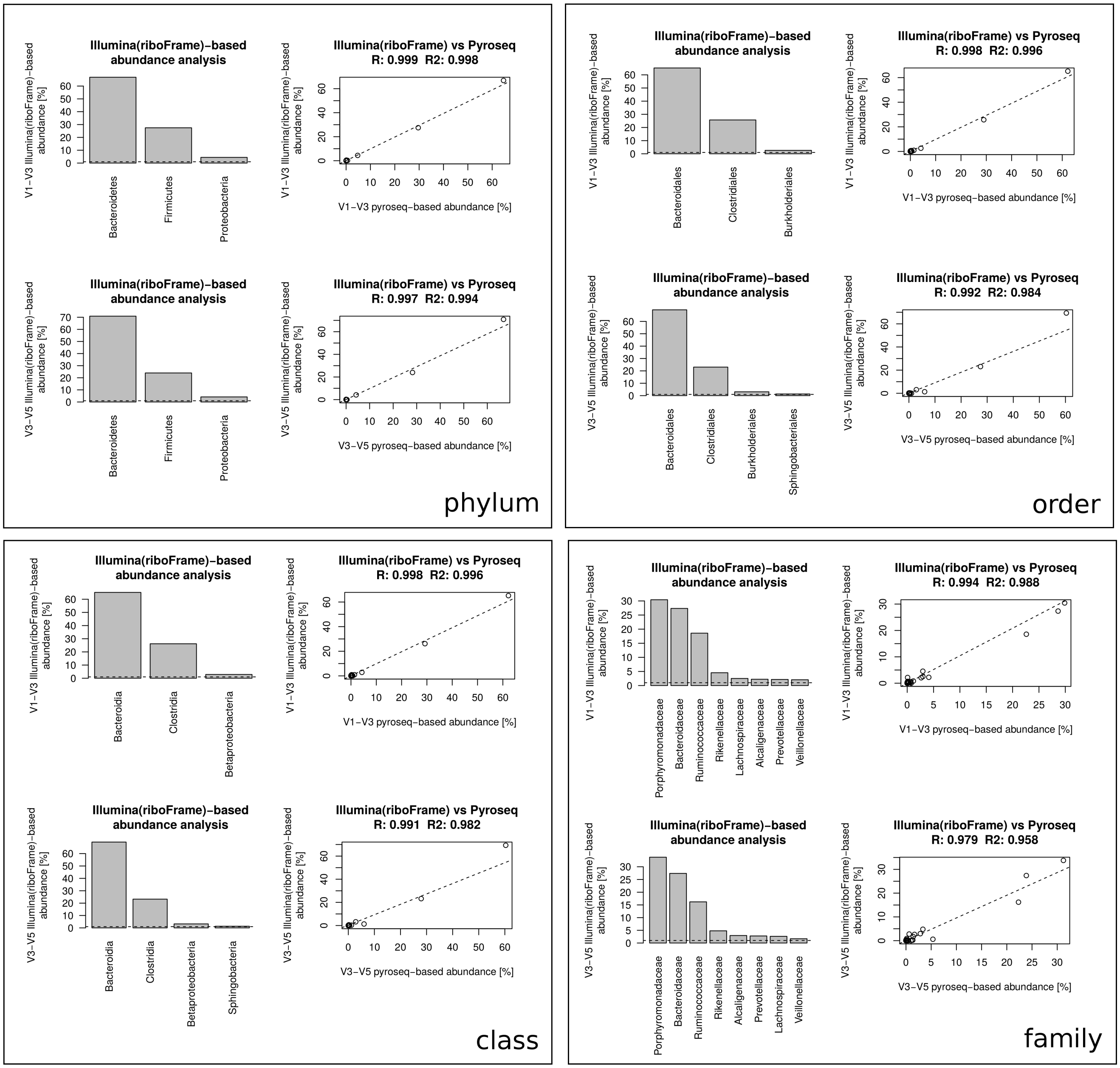

Supplementary figure S5. Variation in taxonomic confidence at different read length for different taxonomic ranks in the HMP sample SRS011061, expressed as quartiles of the confidence score at the different lengths. Black solid, red solid and black dashed lines represent the 75th, 50th and 25th percentile of the distribution of the confidence score at a given read length.

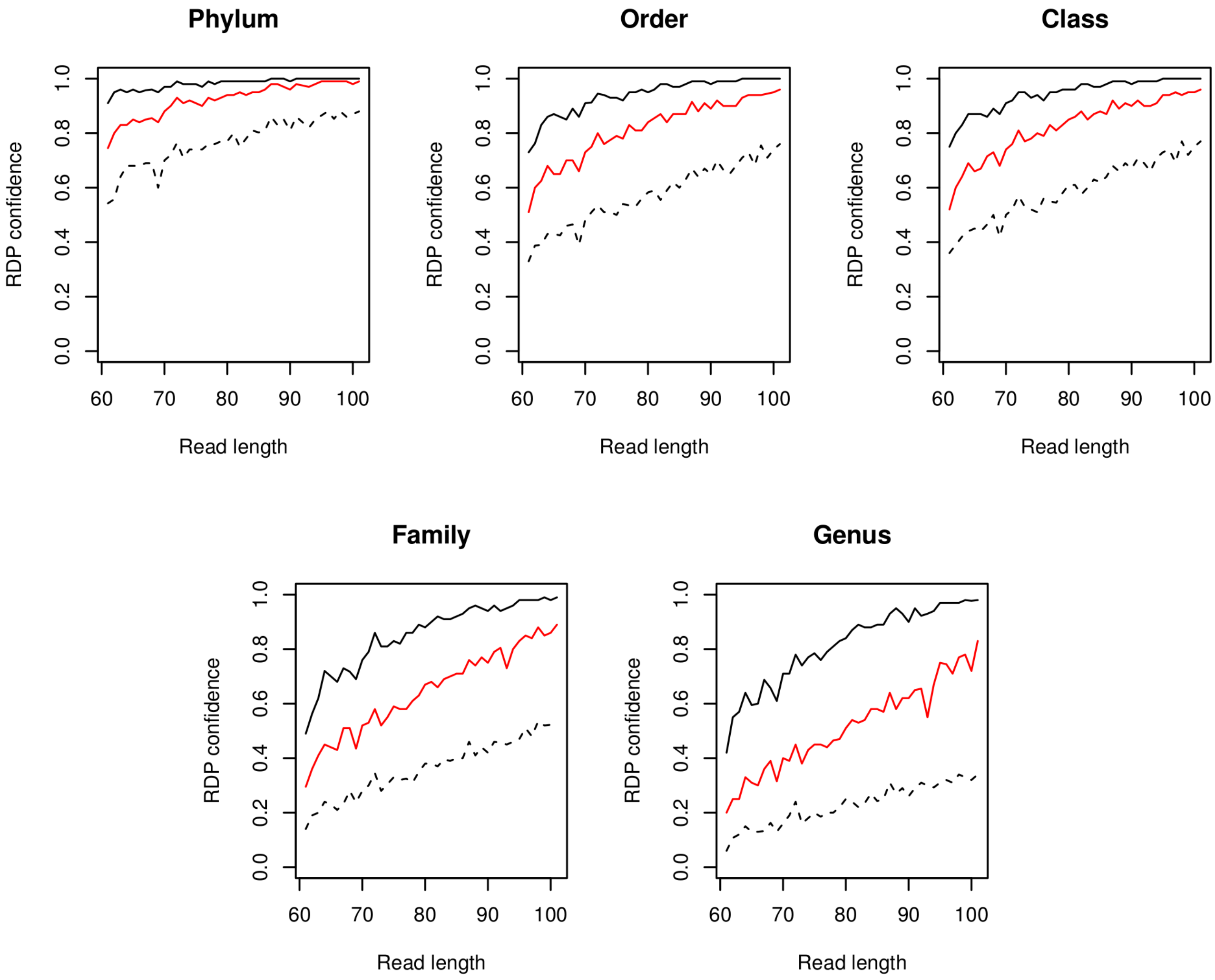

Supplementary figure S6. HMP sample SRS011061. Percent of reads passing an increasing confidence threshold at different taxonomic ranks.

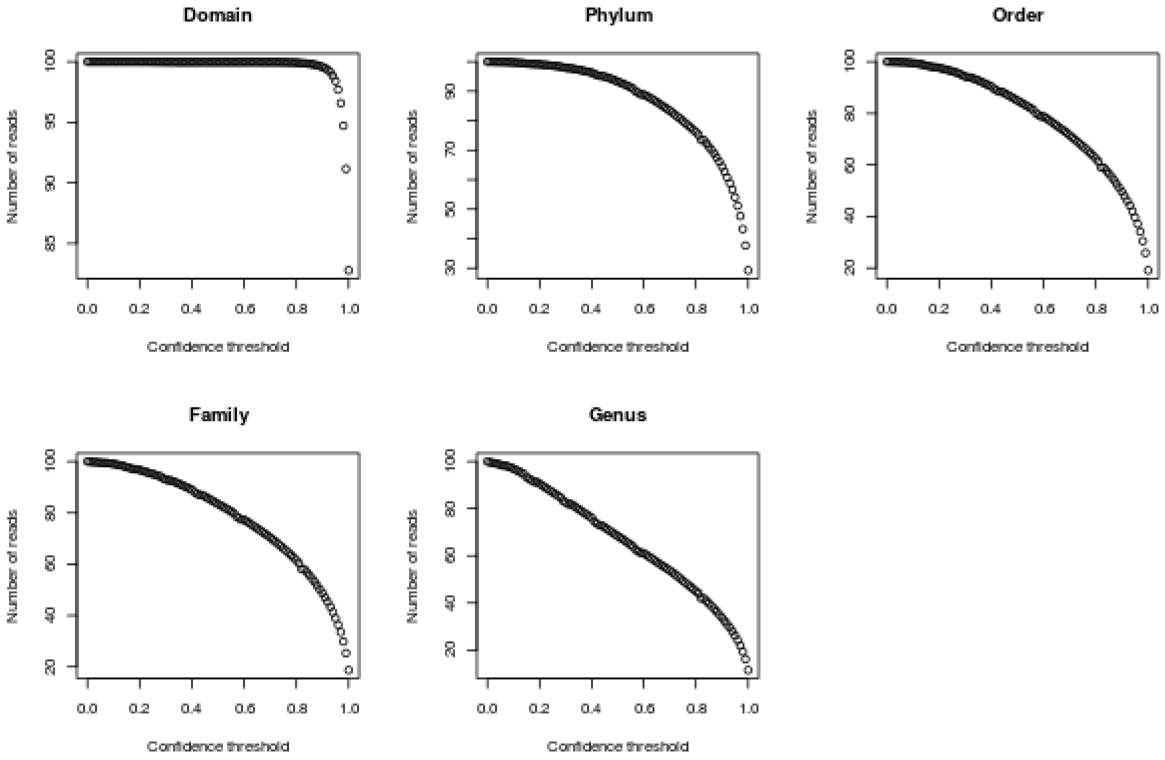

Supplementary figure S7. Distribution of the confidence scores for the genus rank in the 9 variable regions for the HMP sample SRS011061.

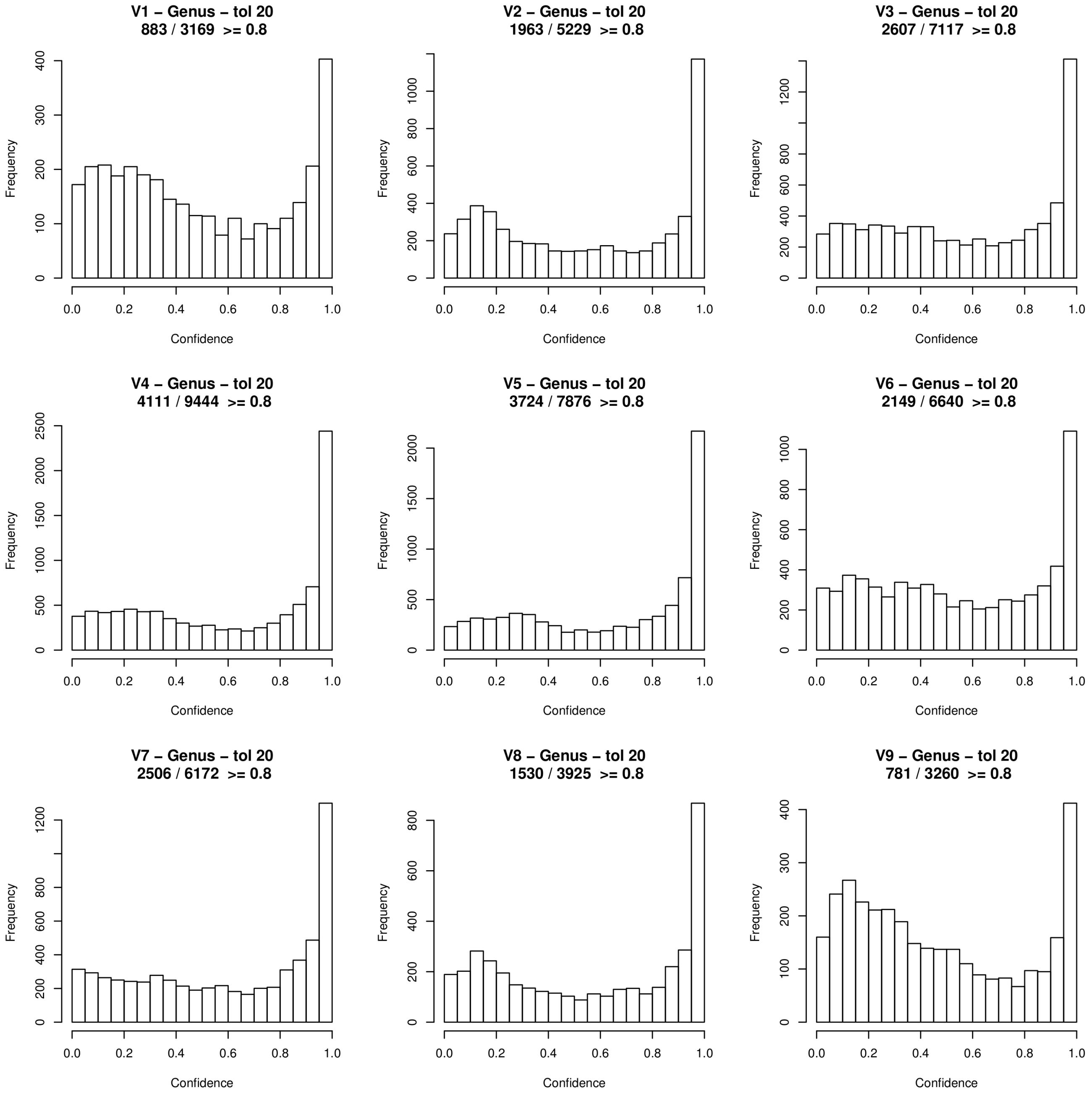

Supplementary figure S8. Results of the EMIRGE analysis on the HMP sample SRS011061. A: Pie chart describing the proportion of identified species. B: Bar plots comparing the proportions (%) of families (top) and genera (bottom) obtained from pyrosequencing of the V3-V5 region, Illumina (riboFrame processed) on the same region and EMIRGE (using the prior abundance columns from the EMIRGE output).

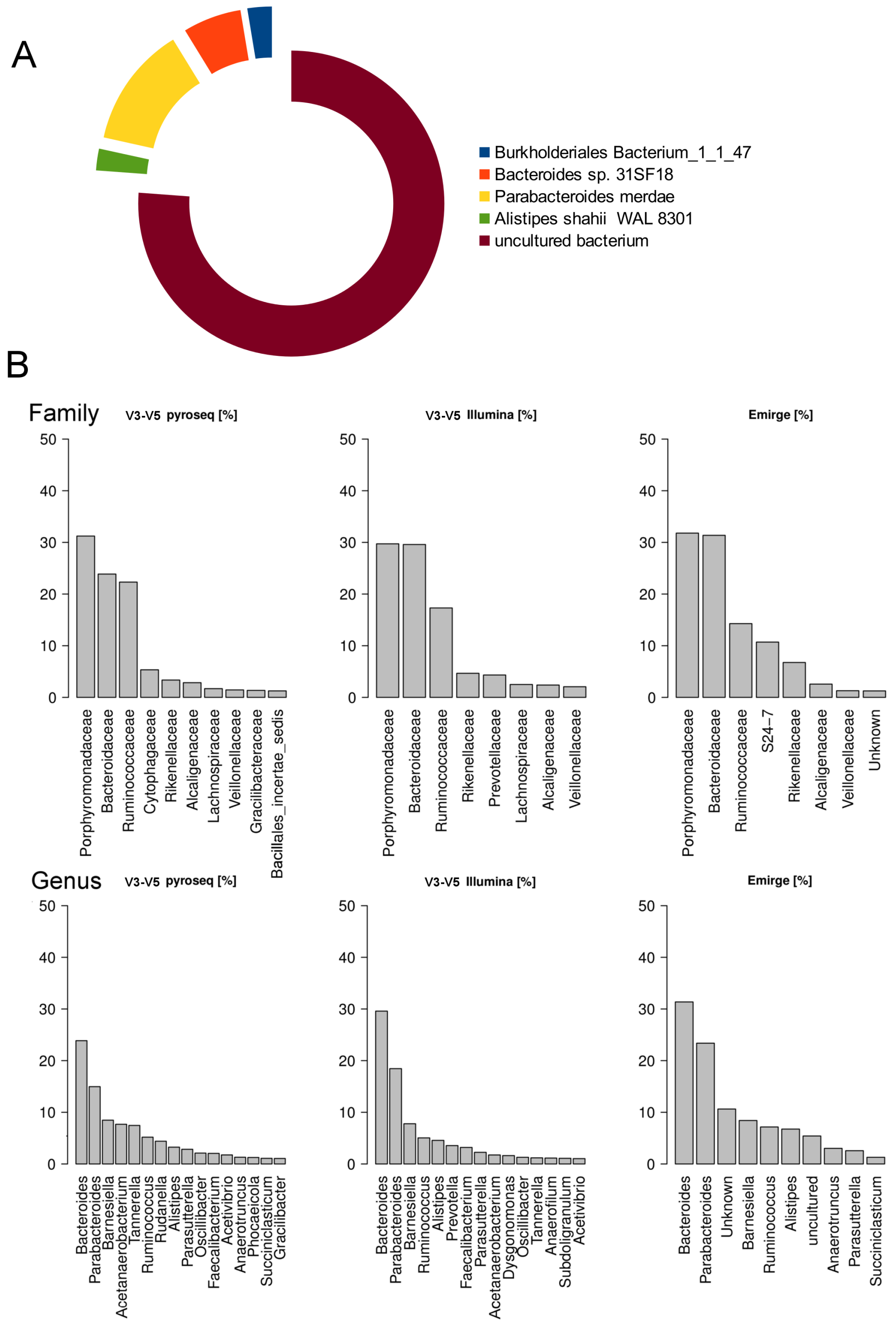

Supplementary data: accession codes and useful links for obtaining or deriving the dataset used in this work (also visit https://github.com/matteoramazzotti/riboFrame for detailed usage instructions and examples)

Simulation of ribosomal reads
Ribosomal 16S sequences for bacteria and archaea were downloaded from
https://rdp.cme.msu.edu/download/current_Bacteria_unaligned.gb.gz
https://rdp.cme.msu.edu/download/current_Archaea_unaligned.gb.gz
Simulation of metagenomics reads with MetaSim
ftp://ftp.ncbi.nlm.nih.gov/genomes/Bacteria/all.fna.tar.gz
ftp://ftp.ncbi.nlm.nih.gov/pub/taxonomy/gi_taxid_nucl.dmp.gz
http://www.plantagora.org/tools_downloads/files/errormodel-100bp.php
Data from Human Microbime Project (HMP)
Sample: SRS011061, human metagenome sample from G_DNA_Stool of a female participant in the e dbGaP study "HMP Core Microbiome Sampling Protocol A (HMP-A)"
v1v3 16S rDNA reads pyrosequencing (454 GS FLX, NCBI SRA download links)
ftp://ftp.ncbi.nlm.nih.gov/sra/sra-instant/reads/ByRun/sra/SRR/SRR056/SRR056886/SRR056886.sra
ftp://ftp.ncbi.nlm.nih.gov/sra/sra-instant/reads/ByRun/sra/SRR/SRR045/SRR055748/SRR055748.sra

Microbial profiling from non-targeted metagenomics

v3v5 16S rDNA reads by pyrosequencing (454 GS FLX, NCBI SRA download links)
ftp://ftp.ncbi.nlm.nih.gov/sra/sra-instant/reads/ByRun/sra/SRR/SRR056/SRR056969/SRR056969.sra
ftp://ftp.ncbi.nlm.nih.gov/sra/sra-instant/reads/ByRun/sra/SRR/SRR055/SRR055668/SRR055668.sra
Metagenomics reads from WGS (Illumina Genome Analyzer II, HMP download link)
http://downloads.hmpdacc.org/data/Illumina/stool/SRS011061.tar.bz2
